# Consecutive Slides on Axial View Is More Effective Than Transversal Diameter to Differentiate Mechanisms of Single Subcortical Infarctions in the Lenticulostriate Artery Territory

**DOI:** 10.3389/fneur.2019.00336

**Published:** 2019-04-09

**Authors:** Yuze Cao, Mengyu Zhang, Lixin Zhou, Ming Yao, Bin Peng, Yicheng Zhu, Jun Ni, Liying Cui

**Affiliations:** ^1^Department of Neurology, Peking Union Medical College Hospital, Peking Union Medical College and Chinese Academy of Medical Sciences, Beijing, China; ^2^Neuroscience Center, Chinese Academy of Medical Sciences, Beijing, China

**Keywords:** single subcortical infarction, lenticulostriate artery territory, etiological categorization, white matter hyperintensity, SMART study

## Abstract

**Objective:** Lipohyalinosis or atherosclerosis might be responsible for single subcortical infarctions (SSIs); however, ways of differentiating between the two clinically remain uncertain. We aimed to investigate whether consecutive slides on axial view or transversal diameter is more effective to differentiate mechanisms by comparing their relationships with white matter hyperintensities (WMHs).

**Methods:** All the participants from the Standard Medical Management in Secondary Prevention of Ischemic stroke in China (SMART) cohort who had SSIs in the lenticulostriate artery territory were included and categorized according to consecutive slides on axial view (≥4 consecutive slices or not) and transversal diameter (≥15 mm or not). The associations between the severity of WMHs and the different categories were analyzed.

**Results:** Among the 3,821 patients of the SMART study, 281 had diffusion-weighted image-proven SSIs in the lenticulostriate artery territory. When classified by consecutive slides on axial view, SSIs on ≥4 slices were significantly associated with the severity of the WMHs, both in deep WMH (DWMH) (odds ratio [OR], 0.32; 95% confidence interval [CI], 0.11–0.97; *p* = 0.04) and periventricular hyperintensity (PVH) (OR, 0.37; 95% CI, 0.17–0.78; *p* = 0.01). No such association was found on the basis of the transversal diameter (*p* > 0.1).

**Conclusion:** Consecutive slides on axial view (≥4 consecutive slices) might be more effective than transversal diameter to identify the atherosclerotic mechanisms of SSIs in the lenticulostriate artery territory.

**Clinical Trial Registration:** http://www.clinicaltrials.gov. Unique identifier: NCT00664846

## Introduction

Single subcortical infarctions (SSIs) have been considered to be caused by lipohyalinosis degeneration in small artery disease, traditionally called lacunar infarct (1). However, atherosclerosis occurring in the parental artery blocking the orifice of the branch artery or atherosclerosis in the proximal branch artery can also contribute to the etiology of SSIs, which are termed as “branch atheromatous disease” (2, 3). Both the “lipohyalinotic” and “atheromatous” mechanisms of SSIs have been pathologically proved (4, 5), but the two are difficult to distinguish *in vivo*. Although computed tomographic angiography (CTA) and magnetic resonance angiography (MRA) are used to detect atherosclerosis frequently, they cannot detect diffuse atheromatous wall involvement without focal stenosis. Current direct and conventional imaging techniques, including high-resolution magnetic resonance imaging (HRMRI) cannot identify the wall features of the branch artery effectively (6), which limits further research on the etiology of SSIs.

Clinically, identification of different etiologies is of great importance in guiding treatment and predicting functional prognosis (3, 7). For SSIs in the pontine, unilateral lesions extending to the ventral surface were considered an atherosclerotic mechanism. However, for SSIs in the lenticulostriate artery territory, methods to differentiate mechanisms are still controversial. One method is based on the diameter of the ischemic lesion, and another is based on the consecutive slides on axial view. Whether the consecutive slides on axial view or transversal diameter is more effective remains controversial. As white matter hyperintensities (WMHs) have been widely considered as the most frequently used imaging marker of cerebral small vessel disease, identifying the different etiological subtypes of SSIs by comparing their discrepancies in WMHs might be helpful. Given the anatomical features of the lenticulostriate artery, we hypothesized that consecutive slides on axial view would be more effective than transversal diameter to predict the mechanism of SSIs. To further clarify this issue, we examined the association between WMHs and the lesion characteristics of SSIs in a prospective multicenter cohort study, known as the Standard Medical Management in Secondary Prevention of Ischemic stroke in China (SMART) study (8).

## Materials and Methods

### Study Population

Data were obtained from the SMART study, a large, multicenter, randomized controlled trial to assess the effectiveness of a guideline-based program in secondary stroke prevention in 47 hospitals in China. The detailed study protocol and main results of the SMART study have been published elsewhere (8, 9). Among the 3,821 participants enrolled in the SMART study between April 2008 and December 2010, 1,129 were proven to have acute ischemic stroke by using diffusion-weighted imaging (DWI). Of the patients, 281 with single subcortical infarctions located in the lenticulostriate artery territory were included. Patients with a probable etiology of cardioembolism or lack of complete images for review were excluded.

Demographic features and risk factors were reviewed from data collected from the database, including age, sex, hypertension, diabetes mellitus, hyperlipidemia, and smoking habit.

The SMART study was approved by the central ethics committee of the leading study center at Peking Union Medical College Hospital and the ethics committees of all participating institutions. Written informed consent was obtained from all the participants or their legal surrogates.

### Neuroimaging

#### White Matter Hyperintensity

WMHs are hyperintense on T2-weighted sequences, isointense or hypointense on T1-weighted sequences, and isointense on diffusion-weighted images (DWI) (10). The burden and severity of WMHs were assessed on the basis of the Fazekas visual rating scale (11). Periventricular hyperintensity (PVH) and deep WMH (DWMH) were scored separately. DWMH was rated as follows: 0 = absence, 1 = punctate foci, 2 = beginning confluence of foci, and 3 = large confluent areas. PVH was rated as follows: 0 = absence, 1 = caps or pencil-thin lining, 2 = smooth halo, 3 = irregular PVH extending into the deep white matter. Scores of 2 and 3 were defined as severe WMHs. All WMHs were independently graded by 2 well-trained neurologists. The inter-rater reliability was assessed using a subset of 50 random study subjects. Kappa value for the inter-rater agreement was 0.85 for PVH and 0.89 for DWMH.

#### Lesion Features

**Figure 1:**
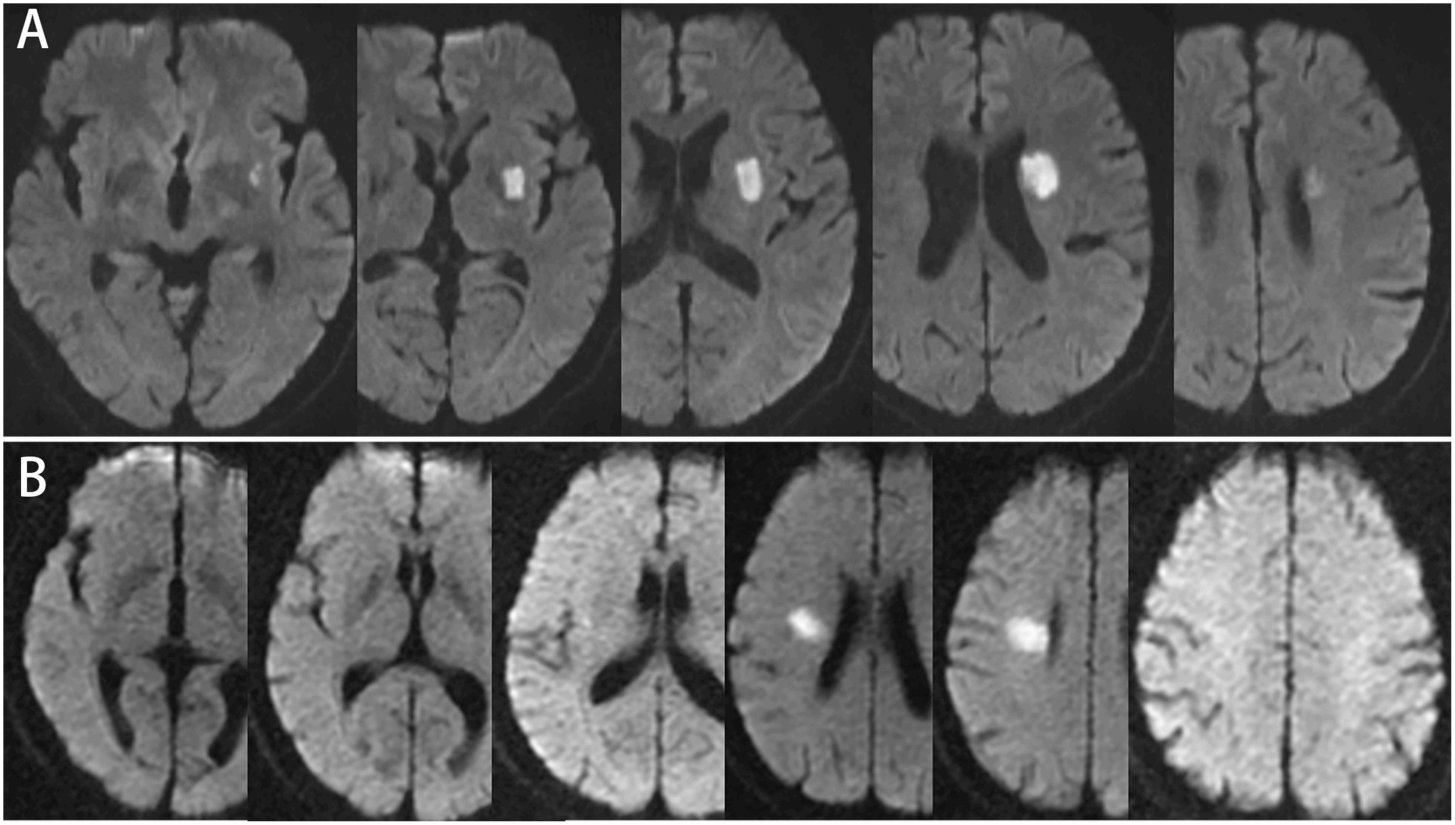
**(A)** 57-year-old male, consecutive slices DWI imaging: infarct lesion ≥4 slices, involving the base of basal ganglia, but the maximum axial diameter is 21.7 mm. **(B)** 52-year-old male, consecutive slices DWI imaging: infarction lesion ≤3 slices, involving radiation corona, and the maximum diameter of axis is 21.2 mm.

In this study, transversal and longitudinal features of single subcortical infarctions pertaining to the lenticulostriate artery terminations (single ischemic lesions located in the lentiform nucleus and/or corona radiate) were analyzed. In previous published studies, two-dimensional cutoffs of lesion features were widely used to differentiate the atheromatous mechanism of SSIs from the traditional lacune (3, 12, 13). Transversally, the lesion ≥15 mm in diameter on the axial picture, or longitudinally, the lesion observed in ≥4 axial pictures (Figure 1) were suggestive of atheromatous mechanisms. In our study, MRI scans were performed using 1.5 or 3.0 T MR imaging units. Slice thickness ranged from 5 to 6 mm in different centers. We defined the following findings on DWI to determine lesion features. “Transversal diameter” was measured as the maximal diameter of the lesion on DWI. “Consecutive slides on axial view” was defined as the number of lesion axial slices visible on DWI to evaluate the longitudinal extension of the lesion. All the SSI lesions were classified on the basis of the two criteria.

### Statistical Analyses

In the description of general clinical characteristics, continuous variables were expressed as mean ± standard deviation (SD) or median (interquartile range). The categorized variables were reported as percentage. To examine the association between the severity of WMHs and SSI features, binary logistic regression models, with adjustment for age, sex, and hypertension, were computed with dichotomized SSI lesion characteristics, using <4 consecutive slides on axial view or a transversal diameter of <15 mm as the reference category. *P*-values of <0.05 for the two-tailed test were considered significant. Statistical analyses were performed using IBM SPSS statistics version 19 (SPSS Inc., Chicago, IL, USA).

## Results

### Demographic Characteristics

Of the 3,821 participants of the SMART study, 281 (7.35%) with DWI-proven SSIs were enrolled in the study. The general characteristics and risk factors of the patients in the two groups are summarized in Table 1. Seventy-four patients (26.33%) with SSIs had ≥4 consecutive slices, and 137 patients (53.94%) had a diameter of ≥15 mm.

**Table 1:** General characteristics and vascular risk factors.

	**Consecutive slides on axial view**		**Transversal diameter**	
	**<4 slices**	**≥4 slices**	**<15 mm**	**≥15 mm**
Total number	207/281	74/281	117/254	137/254
Age (mean ± SD), years	63.40 ± 11.73	57.31 ± 10.39	64.81 ± 11.34	58.86 ± 11.37
Sex (male), %	144 (69.2)	49 (66.2)	83 (70.9)	94 (68.6)
HTN history, *n* (%)	131 (63.0)	40 (54.1)	74 (63.2)	79 (57.7)
DM history, *n* (%)	42 (20.2)	15 (20.3)	23 (19.7)	26 (19.0)
CI history, *n* (%)	43 (20.7)	9 (12.2)	29 (24.8)	16 (11.7)
HL history, *n* (%)	31 (14.9)	9 (12.2)	17 (14.5)	15 (10.9)
Current smoker, *n* (%)	78 (37.7)	28 (37.8)	67 (57.3)	70 (51.1)
DWMH (≥2)	47 (22.6)	4 (5.4)	29 (24.8)	15 (10.9)
PVH (≥2)	86 (41.3)	11 (14.9)	47 (40.2)	38 (27.7)

*HTN, hypertension; DM, diabetes mellitus; CI, cerebral infarction; HL, hyperlipidemia; DWMH, deep white matter hyperintensity; PVH, periventricular hyperintensity*.

### Associations Between WMHs and SSI Lesion Features

When classified according to consecutive slides on axial view, SSIs of ≥4 slices were significantly negatively associated with both DWMH (odds ratio [OR], 0.32; 95% confidence interval [CI], 0.11–0.97; *p* = 0.04) and PVH (OR, 0.37; 95% CI, 0.17–0.78; *p* = 0.01) after adjustment for age, sex, and hypertension. By contrast, transversal SSI lesion feature, based on a transversal diameter of ≥15 mm, was not found to be statistically related to either DWMH or PVH (as shown in Table 2).

**Table 2:** Associations between WMHs and SSI lesion features.

	**Consecutive slides on axial view**		**Transversal diameter**	
	**≥4 slices vs. < 4 slices**	***P*-value**	**≥15 mm vs. < 15 mm**	***P*-value**
Severe DWMH: Fazekas ≥2	OR, 0.32; 95% CI, 0.11–0.97	0.04	OR, 0.80; 95% CI, 0.46–1.39	0.43
Severe PVH: Fazekas ≥2	OR, 0.37; 95% CI, 0.17–0.78	0.01	OR, 1.02; 95% CI, 0.65–1.58	0.95

*Model: adjusted for age, sex, and hypertension*.

*SSI, single subcortical infarction; WMH, white matter hyperintensity; DWMH, deep white matter hyperintensity; PVH, periventricular hyperintensity; OR, odds ratio; CI, confidence interval*.

## Discussion

The present results obtained in a multicenter cohort with SSIs in the lenticulostriate artery territory showed that SSI lesions of ≥4 consecutive slices negatively correlated with the severity of WMHs, but no such relationship was found in SSI lesions with diameters of ≥15 mm as compared with those with diameters of < 15 mm. Our findings strongly suggest that consecutive slides on axial view might be more effective than transversal diameter for identifying the etiologies of SSIs in the lenticulostriate artery territory.

The etiologies and mechanisms of SSIs have been a research focus in recent years. The mechanisms could be classified as atherosclerotic and lipohyalinotic (6), and the former could be further categorized into three groups as follows: plaque of the parental artery obliterating the orifice of the branch artery, plaque from the parental artery extending into the branch artery, and microatheroma blocking the proximal portion of the branch artery (2). Differentiation of the atherosclerotic and lipohyalinotic mechanisms of SSIs is highly important because of their different prognoses and treatment strategies (3, 7, 12). This would be helpful to guide the treatment with dual or single antiplatelet therapies in the acute phase and secondary prevention. SSIs with atherosclerotic causes are more prone to early neurological deterioration (END) (7), and for these patients, dual antiplatelets and intensive statin therapy might be necessary.

As described previously, the conventional imaging technique cannot be used in the direct diagnosis of the etiology of SSIs, especially those in the proximal portion of the branch artery (6). HRMRI was found to be useful in detecting atherosclerotic plaques in medium-to-large vessels (14, 15). However, all the above-mentioned methods cannot visualize atherosclerosis in the proximal branch artery. Therefore, the mechanisms of SSIs cannot be identified effectively using direct and conventional imaging techniques, which limits clinical decision.

Previous studies of subtentorial SSIs mainly focused on pontine infarction and supratentorial SSIs on lenticulostriate artery territory (3). As for pontine infarctions, unilateral lesions extending to the ventral pontine surface were usually considered as atherosclerotic mechanisms (16–18). However, as for SSIs in the lenticulostriate artery territory, approaches to differentiate mechanisms still remain controversial. Previous studies suggested that the diameter of the SSI lesion could indicate the underlying mechanisms, as SSIs with atheromatous mechanisms are expected to be larger than lacunar infarcts (7, 12). However, others believe that consecutive slices on a transversal plane might differentiate the mechanism, as atheromatous lesions are located proximally along the branch artery (13, 19). The more appropriate method must be further clarified.

In the present study, SSIs with ≥4 consecutive slices, rather than a diameter of ≥15 mm, were found to negatively correlate with WMHs. As WMH is widely considered to be a strong imaging marker of small vessel disease (SVD), SSIs caused by atherosclerotic disease might have less severe WMHs than those caused by SVD. Thus, our findings suggest that classifying SSIs according to ≥4 consecutive slices might be more effective for identifying the atherosclerotic mechanisms of SSI. This could be explained by the anatomy of the branch artery. Findings from a recent 7-T MRI research showed that SSIs caused by lenticulostriate artery occlusion were more likely to extend from the corona radiata to the lower part of the basal ganglia along the longitudinal direction, which was compatible to the distribution of the lenticulostriate artery (20). More than 4 consecutive slices implies that the lesion involves the base of the basal ganglia and is closer to the orifice of the lenticulostriate branch artery. While atherosclerotic vascular lesions are located proximally along the branch artery in comparison with lipohyalinosis, the etiology of SSIs of ≥4 consecutive slices are more likely to be atherosclerotic at the orifice of the branch artery or its parent artery. Furthermore, considering the possible deviation of the diameter measurement, consecutive slides on axial view are easy to evaluate clinically.

The methodological strengths of our study include its prospective multicenter design and the large sample size of the SMART cohort, even though the subgroup analysis was retrospective. In addition, confirmation of different mechanisms requires autopsy, which is difficult to attain. In this study, we provided a new perspective by using WMHs to compare the effectiveness of different lesion features. However, our study has several limitations. First, the variability of the MRI parameter, including the field strength and thickness of the considered slice, among the 47 participating hospitals in the SMART study may have a potential influence on the description of ischemic lesions. However, the MRI slice thickness in most hospitals was 6 mm, which minimizes the difference among the centers. Second, the mechanism was not confirmed, as our findings provided only circumstantial rather than direct evidence for differentiating the etiologies and mechanisms of SSIs. Recently, 7-T MRI has been shown to be sensitive for identifying small branches (20), further research studies are needed to confirm this.

## Conclusion

In summary, the present study suggests that consecutive slides on axial view (≥4 consecutive slices) might be more effective than transversal diameter for identifying the mechanisms of SSIs in the lenticulostriate artery territory. To establish the association between the lesion features and mechanisms of SSI, future investigation with a 7-T MRI device might be necessary.

## Author Contributions

YC and JN contributed to the writing of the article. MZ, MY, and YZ contributed to the collection of data. LZ and JN contributed to the rating of images. LC and BP contributed to revising the article. All authors read and approved the manuscript.

### Conflict of Interest Statement

The authors declare that the research was conducted in the absence of any commercial or financial relationships that could be construed as a potential conflict of interest.
